# Estimation of gestational age from fundal height: a solution for resource-poor settings

**DOI:** 10.1098/rsif.2011.0376

**Published:** 2011-08-17

**Authors:** Lisa J. White, Sue J. Lee, Kasia Stepniewska, Julie A. Simpson, Saw Lu Mu Dwell, Ratree Arunjerdja, Pratap Singhasivanon, Nicholas J. White, Francois Nosten, Rose McGready

**Affiliations:** 1 Centre for Clinical Vaccinology and Tropical Medicine, Nuffield Department of Clinical Medicine, John Radcliffe Hospital, University of Oxford, Oxford OX3 7LJ, UK; 2 Faculty of Tropical Medicine, Mahidol University, Bangkok 10400, Thailand; 3 Centre for Molecular, Environmental, Genetic and Analytic Epidemiology, School of Population Health, The University of Melbourne, Melbourne, Victoria 3010, Australia; 4 Shoklo Malaria Research Unit, PO Box 46 Mae Sot, Tak 63110, Thailand

**Keywords:** symphysis-fundal height, gestational age, estimation, formula, ultrasound

## Abstract

Many women in resource-poor settings lack access to reliable gestational age assessment because they do not know their last menstrual period; there is no ultrasound (US) and methods of newborn gestational age dating are not practised by birth attendants. A bespoke multiple-measures model was developed to predict the expected date of delivery determined by US. The results are compared with both a linear and a nonlinear model. Prospectively collected early US and serial symphysis-pubis fundal height (SFH) data were used in the models. The data were collected from Karen and Burmese women attending antenatal care on the Thai–Burmese border. The multiple-measures model performed best, resulting in a range of accuracy depending on the number of SFH measures recorded per mother (for example six SFH measurements resulted in a prediction accuracy of ±2 weeks). SFH remains the proxy for gestational age in much of the resource-poor world. While more accurate measures should be encouraged, we demonstrate that a formula that incorporates at least three SFH measures from an individual mother and the slopes between them provide a significant increase in the accuracy of prediction compared with the linear and nonlinear formulae also using multiple SFH measures.

## 1. Introduction

Ultrasound (US) assessment of gestational age up to 24 weeks provides the most accurate prediction of the expected date of delivery (EDD) and is more reliable than the last menstrual period (LMP) [1,2]. Although accurate gestational age assessment is not a problem unique to resource-poor settings [3–5], there is lower availability of US dating for women in these settings [6–8]. Owing to the sheer numbers of births and economics in developing countries, the LMP remains to be the most widespread predictor of gestational age [9,10]. In some cultures, particularly where literacy levels are low, LMP can be very unreliable [7]. In such settings, methods to date such pregnancies have relied on inexpensive tools including validated scored assessments of superficial and neurological newborn criteria, for example the Dubowitz [4,11–13] and Ballard or modified Ballard [4,14–18] score. Training and ongoing quality control of testers are needed to maintain the accuracy of these methods. The symphysis-pubis fundal height (SFH) measurement is also widely available, routinely practised in nearly all antenatal settings in the world and simple to perform. While Neilson's [19] Cochrane review concludes that there is not enough evidence to evaluate the use of SFH during antenatal care (ANC), it may be the only data collected and reported in an antenatal card, in much of the resource-poor world, which provides a clue to the gestation of pregnancy. In the past 20 years, SFH has taken a back seat to US in terms of gestating pregnancies but resource-rich [20–25] and -poor [8,18,26–28] countries use SFH in routine practice as a low technology method for monitoring foetal growth and identifying intrauterine growth restriction.

Attempts have been made to use SFH and other factors such as maternal weight and US prediction to infer excess foetal weight with moderate success [29–31]. A single SFH at delivery was not reliable enough to estimate foetal weight in South Africa [29–32] but was felt to be useful in rural Tanzania [33]. SFH after 24 weeks has been used to schedule the start of zidovudine therapy to prevent mother-to-child-transmission of HIV when LMP or US were not available or reliable [8].

In one UK-based study, an obstetrician blinded to the LMP overestimated gestation by six weeks when assuming SFH at the umbilicus was equivalent to 20 weeks [34]. SFH has been used as a proxy for gestational age in Africa [26] and racial differences in SFH growth rates have also been documented [35,36]. Crosby & Engstrom [37] and Engstrom *et al*. [38,39] emphasize the considerable inter- and intra-observer error in their study of SFH measurements. The shape of the SFH curve with gestation has been plotted by various groups who established population curves again in the interests of being able to detect growth restriction [35,38,40–46]. Two of these groups describe the use of polynomial regression as the best method to fit the SFH data [41,45]. Few studies have modelled SFH to predict gestational age at birth [26].

In refugee camps and migrant antenatal clinics on the Thai–Burmese border, the majority of women are unable to provide a reliable date of the LMP [7]. In previous publications on malaria in pregnancy from the same area, a formula for predicting gestational age using SFH in these women was used [47,48] and was found to predict gestational age with an accuracy of ±6.26 weeks.

Variations in foetal size at a given gestation can be converted into differences in gestational age. This applies just as well to US estimates (current gold standard) though this is rarely discussed [49]. Henriksen *et al*. [49] explored this in detail in relation to good quality history of LMP and an early US measurement of early biparietal diameter (BPD) in 3606 women. They report that factors that reduce foetal size, e.g. female sex of babies and maternal smoking, can distort the relative risk of preterm or post-term delivery by 10–20 per cent when gestational age is based on late US not LMP. Despite highly accurate foetal measurements at present, an inherent error remains in any prediction of gestational age. This paper refines the estimation of gestational age from SFH in women using early US-derived gestation as a gold standard. Three models (formulae) were developed and compared for accuracy of predictive power. The aim of modelling SFH in this particular population was to ascertain the most reliable method of gestating pregnancies when no other reliable measure of gestation was available.

## 2. Methods

### 2.1. The data

Shoklo Malaria Research Unit (SMRU) is located on the Thai–Burmese border and has studied the epidemiology, prevention and treatment of malaria in pregnancy since 1986. It has five established clinics, one of which is based in Maela refugee camp, where the Karen minority group from Burma are the principal inhabitants. In all of its clinics, SMRU runs a programme of ANC to detect and treat all parasitaemic episodes during pregnancy through weekly malaria screening in order to prevent maternal death [50]. Since the inception of this ANC programme, all pregnant women have been encouraged to attend as early as possible during pregnancy. At the first visit (usually between eight and 14 weeks' gestation), US is used to determine viability, detect multiple pregnancy and estimate gestational age. A second scan is performed at 18–24 weeks to confirm gestation, viability and placental position. As this is the only antenatal and delivery service easily accessible to women in these areas, all records are filed in a manner similar to a hospital archive. Patient files are computerized and can be retrieved as needed. Post-term pregnancies are managed by induction. At the time of data collection, the upper limit to commence induction was 42 weeks. Patients were also included in the management plan and some women refused induction.

Anonymous data from pregnancies with live born, congenitally normal, singleton outcomes were collated. The serial SFH measurements (centimetre) and their respective date of measurement in mothers with pregnancies dated by ultrasonography between 8 + 0 to <11 + 0 weeks (crown rump length measured) and 16 + 0 to <21 + 0 weeks (BPD, femur length and abdominal circumference measured) were included in a database. The period of data collection was from April 2002 to May 2006. Women with fewer than three serial SFH measurements or SFH measurements that were less than two weeks apart were also excluded.

SFH was examined in every woman on a weekly basis until it was first measured. SFH was then performed at least monthly and often weekly from 34 weeks onwards. After making sure the bladder was empty, the woman lay down on her back, while the midwife, sitting to the patient's right, located the symphysis pubis. The metal tip (at 0 cm) of a standard soft tape measure (manufactured by Butterfly in the People's Republic of China) was placed at the upper border of the symphysis pubis. SFH was the distance measured from the top of the symphysis pubis to the depression in front of the pad of the middle finger marking the top of the uterine fundus, in the midline of the woman's abdomen. Measures were rounded to the nearest centimetre. Midwives recorded SFH into the antenatal record to the nearest round number, i.e. if greater than or equal to 0.5, the fundal height measurement was rounded up and if less than 0.5 was rounded down, and only at that point it was compared with the US gestation for patient care.

Variables that described the date of the first antenatal consultation, the date of birth, maternal age, gravidity and parity, weight, height and body mass index (measured at the first consultation date), smoking during pregnancy and documented *Plasmodium falciparum* and *P. vivax* malaria during pregnancy were also collated.

### 2.2. Models

Three models were considered for the prediction of gestational age using SFH measurement. The first was a linear formula using a single SFH measure, the second was a nonlinear formula using a single SFH measure and the third was a formula that used multiple measures of SFH combined with the dates of each measurement.

Model 1 requires only a single measure of SFH and uses linear regression to model the gestational age. This is the standard linear formula [48] based on a linear relationship between Dubowitz gestational age assessment [11] and SFH measurements (*n* = 100 women with normal pregnancies).
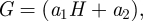
where *G* is the expected gestational age in weeks determined by US at the date of the SFH measurement and *H* is the SFH in centimetres with two estimated parameters *a*_*i*_. This model was transformed to a multiple-measures model by, for each mother, taking the mean of the gestational age at birth predictions from each of her SFH measures.

Model 2 is a nonlinear formula for predicting gestational age. A nonlinear formula was considered because when SFH is plotted against gestational age at the time of measurement for each mother, growth appears to be initially linear followed by a plateau. A functional form was chosen that would allow such a shape while limiting the number of parameters to be estimated to only three.
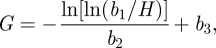
where *G* is the gestational age in weeks and *H* is the SFH in centimetres with three estimated parameters *b*_*i*_. This model was transformed to a multiple-measures model by, for each mother, taking the mean of the gestational age at birth predictions from each of her SFH measures.

Model 3 is a multiple-measures algorithm as follows:

1. Start with a list of SFH with the date they were measured for each mother.
2. Generate all the ‘sets of three’ of these measures. For example, five measures would result in six ‘sets of three’ measures: ([1,2,3], [1,2,4], [1,2,5], [2,3,4], [2,3,5], [3,4,5]).
3. For each ‘set of three’ measures from each mother, predict the gestational age predicted at the final measure for that mother in two ways:
  - Mean of three linear models. 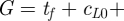 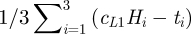.
  - Combination of three fundal heights and three gradients. 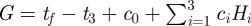

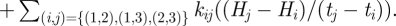
4. If the gestational age predicted by equation (3b) is between *G*_min_ and *G*_max_, then use this for time *t*_*i*_, otherwise use the prediction using equation (3a).
5. For each mother, take the mean of the gestational age predictions for each set of three measures.

This system has 11 estimated parameters: *c*_*L*__0_, *c*_*L*1_, *c*_0_, *c*_1_, *c*_2_, *c*_3_, *k*_12_, *k*_13_, *k*_23_, *G*_min_ and *G*_max_.

### 2.3. Fitting method and model comparison

Chi-squared was used as a measure of goodness of fit and the *χ*^2^ value was minimized within Excel using the simplex method. This approach represents a weighted least-squares minimization where the mean is a proxy for the variability. A subset of the data was produced by randomly selecting the data of 50 per cent of the mothers included in the study population. The model was fit to this subset and then used to predict the gestational age for the remaining data. Results for the predictions of gestational age by USs and models 1–3 in the form of:

— A: Relative percentages of predicted premature (<37 weeks' gestation), term (37 to <42 weeks' gestation) and post-term (≥42 weeks' gestation) births.
— B: The model's potential to predict premature births in the form of a table of true positive; true negative; false positive; false negative; sensitivity; specificity.
— C: The model's potential to predict post-term births in the form of a table of true positive; true negative; false positive; false negative; sensitivity; specificity.
— D: Histogram of predicted gestational age at birth.
— E: Histogram of residual error (i.e. how does the model prediction using only SFH compare with the US prediction—for a good model, the distribution should be symmetrical about zero and have a small spread).
— F: Mean residual error.
— G: Percentage born within two weeks of predicted date of birth.

Results were calculated for the following:

— US data (gold standard thus no result for D).
— Model 1 (linear model):
  - On first SFH.
  - On mid SFH.
  - Average prediction from all SFH.
— Model 2 (nonlinear model):
  - On first SFH.
  - On mid SFH.
  - Average prediction from all SFH.
— Model 3 (multiple-measures model).

### 2.4. Risk factors

For model 3, the predicted gestational age was adjusted for mother-level factors (each of: mother weight; mother height; mother BMI; mother age; the gravida of the current pregnancy; the parity of the current pregnancy; whether the mother smoked or not; slide positive for *P. falciparum*; slide positive for *P. vivax*). Each of these factors was checked for statistical significance using the *χ*^2^ distribution to compare the baseline model fit with that including adjustment by each risk factor.

## 3. Results

### 3.1. Preliminary data analysis

**Figure 1.**
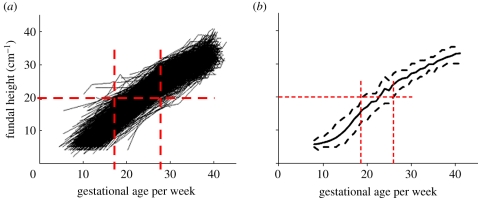
(*a*) Plot of symphysis-fundal height (SFH) against gestational age estimated using ultrasound for all mothers in the study. (*b*) Plot of the mean SFH (solid black line) for each gestational age with 10th and 90th percentiles (dashed black line). Both graphs show the variation associated with an SFH of 20 cm (dashed grey/red online). (Online version in colour).

**Figure 2.**
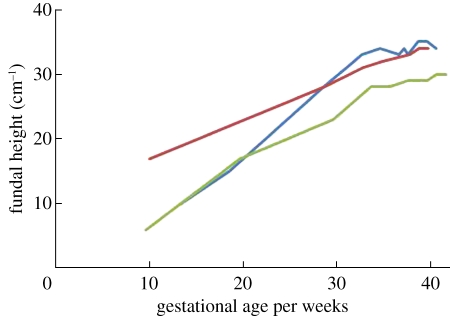
Plot of three example mothers to demonstrate the variation in symphysis-fundal height growth rates at the mother level. (Online version in colour.)

Overall 2437 women with US-dated pregnancies had a total of 7476 SFH measurements with their corresponding dates. The demographic variables of the women included in the model were summarized (table 1). For each mother, the series of SFH measurements was plotted against the gestational age, inferred from the best (crown rump length preferred over BPD) single US estimate at the time of measurement (figure 1). There was a large variation in gestational age for a single SFH (about 10 weeks, figure 1). The variation in SFH for a given gestational age was a combination of the variation between measurements and the variation between individuals. Each mother has a different growth pattern for SFH versus gestational age (figure 2). For example, a plot of the profiles for three mothers (figure 2) shows that the profiles for each mother can be quite different in shape. Two mothers (blue and green) have a similar fundal height early in their pregnancies but have significantly diverged throughout the pregnancies, whereas another mother (red) has a much higher fundal height early in her pregnancy but the growth is slower and converges to the blue line towards the end of the pregnancy. Hence, the challenge in estimating gestational age from multiple measures of SFH in a single woman was to develop a method to accurately account for the placement of an individual growth curve on the gestational age axis.

**Table 1.** The demographic variables of the refugee and migrant women. Numbers expressed as mean ± s.d. (min–max) or % proportion (*n*). CRL, crown rump length; BPD, biparietal diameter; EGA, estimated gestational age.

	*n*	
age (years)	2437	26.5 ± 6.6 (15–48)
weight (kg)	2435	48 ± 7 (30–90)
height (m)	1888	1.51 ± 0.53 (1.30–1.68)
BMI	1887	20.9±2.8 (12.7–36.5)
gravida (median)	2437	3 (1–15)
parity (median)	2437	2 (0–13)
primigravida, % (*n*)	2437	19.5 (480)
smokers, % (*n*)	2421	30.2 (735)
*Plasmodium falciparum*, % (*n*)	2437	3.9 (95)
*Plasmodium vivax*, % (*n*)	2437	9.3 (226)
SFH measurements	2437	7 (2–16)
EGA by CRL%	2437	67.8 (1652)
EGA by BPD%	2437	32.2 (785)

### 3.2. Parameter estimates

For each model, the following parameter values were estimated using the method described earlier:

— Model 1: *a*_1_ = 4.5 and *a*_2_ = 1.0.
— Model 2: *b*_1_=53.96, *b*_2_ = 0.055 and *b*_3_ = 24.82.
— Model 3: *c*_*L*0_ = 4.1, *c*_*L*1_ = 0.95, *c*_0_ = 12, *c*_1_ = 1.1, *c*_2_ = 0.24, *c*_3_ = −0.7, *k*_12_ = 0.09, *k*_13_ = 7, *k*_23_ =−0.1, *G*_min_ = 33 and *G*_max_ = 42.

The electronic supplementary material includes full details of all the model fits. Model 3 was the best-fitting model most closely mimicking the distribution of gestational age at birth as predicted by US and with the lowest variance in residual error.

The 95 per cent prediction interval was calculated for the predictions of gestational age by model 3 for mothers with three to greater than or equal to 10 measurements (table 2) demonstrating that the accuracy of the prediction was not improved using more than six measurements. Six to seven SFH measurements produced 95 per cent prediction intervals of (−16 to 14) and (−15 to 16) days (table 2).

**Table 2.** The 95% prediction interval in days for model 3 predictions according to the number of SFH measurements. Estimates were derived from the model prediction of the 50% of the data not used to fit the model.

number of fundal height measurements (Ge)	3	4	5	6	7	8	9	≥10
lower limit	−36	−30	−21	−16	−15	−15	−12	−11
upper limit	29	21	16	14	16	18	18	21

The predicted gestational age was adjusted for mother-level factors (each of: mother weight; mother height; mother BMI; mother age; the gravida at the current pregnancy; the parity of the current pregnancy; whether the mother was primigravida or not; whether the mother smoked or not; slide positive for *P. falciparum*; slide positive for *P. vivax*) and inclusion of these factors was checked for significance using the *χ*^2^ distribution. The inclusion of any of the mother-level factors does not significantly improve the fit of model 3 most probably because most pregnancies are in normal healthy non-smoking multi-gravid women.

Model 3 was used to explore the cutoff for gestational age for optimal prediction of premature births (table 3). For predicting a premature birth correctly, increasing the cutoff increases sensitivity at the expense of specificity. A cutoff of 37.6 will give a high sensitivity with more true positives and true negatives and a much lower ratio of true to false positives than that given by the standard cutoff of 37 weeks. This indicates that while the use of model 3 with an adjusted cutoff for defining a premature birth is the most effective model for defining premature birth, the ranges defining premature (less than 37 weeks' gestation), term (37 to less than 42 weeks' gestation) and post-term (greater than or equal to 42 weeks' gestation) births in this dataset were approximately five, five and two weeks, respectively. These ranges are small and very similar to the best prediction interval (associated with mothers with many SFH measurements).

**Table 3.** Exploration of the relationship between the cutoff gestational age for defining premature birth and the predictive power of the model for this category. Estimates were derived from the model prediction of the 50% of the data not used to fit the model.

cutoff	positive predictive value (%)	negative predictive value (%)	sensitivity (%)	specificity (%)
37	52	97	46	97
37.2	50	97	50	97
37.4	45	97	57	96
37.6	40	98	62	94
37.8	34	98	65	92
38	29	98	66	90
38.2	25	98	71	87
38.4	21	98	74	83
38.6	18	98	77	78
38.8	15	98	79	72
39	13	98	81	66

**Figure 3.**
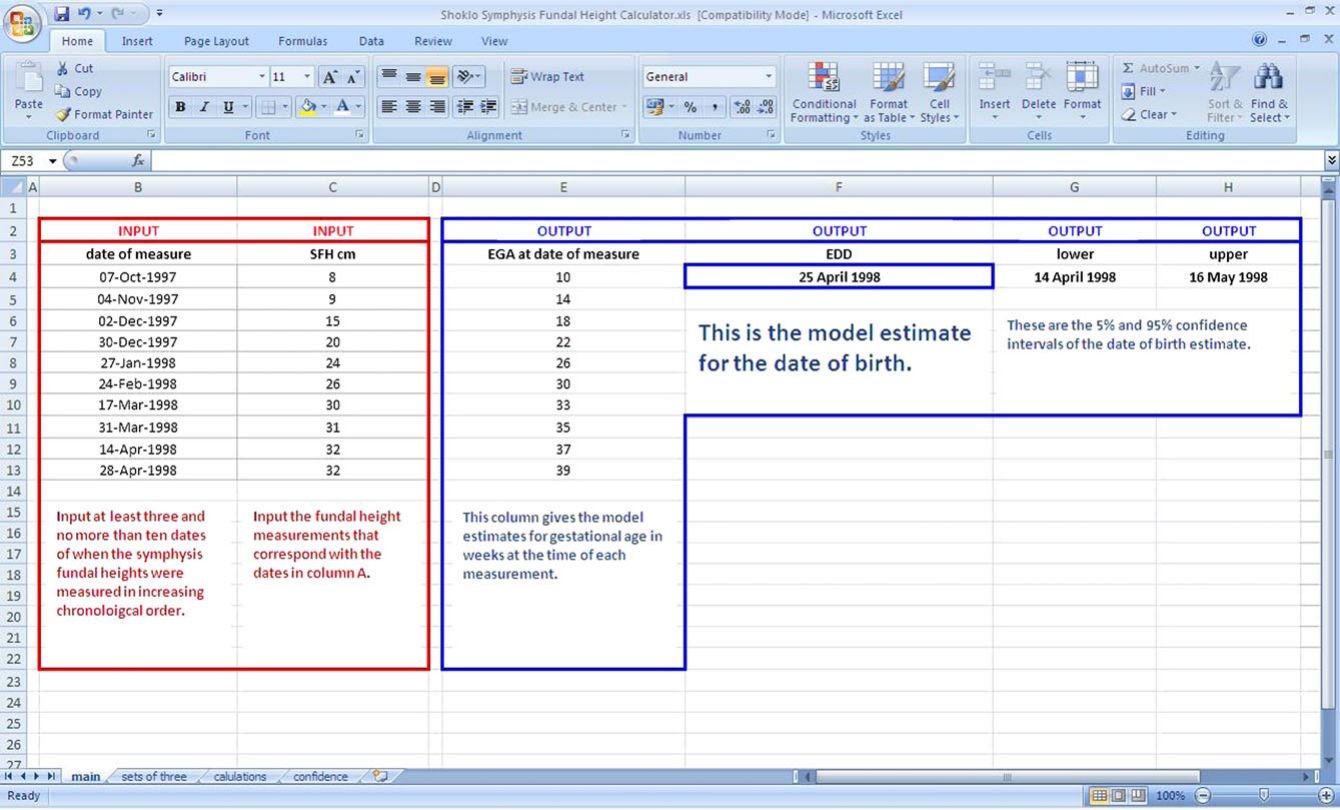
A screenshot of the Shoklo symphysis-fundal height calculator. (Online version in colour.)

We have produced a file within Excel that uses model 3 to estimate gestational age (figure 3). It requires a minimum of three input values of SFH with the dates of measurement. This can be downloaded free of cost from http://www.tropmedres.ac/research/mathematical-modelling/gestational-age.html for use on personal computers.

## 4. Discussion

There have been many publications of SFH-based foetal growth curves in the literature [27,51,52], including two from Thailand [42,53]. We have derived a similar growth curve for the data presented here (figure 1*b*). The inherent variability of the SFH measurement observed in this dataset was a gestational age range of about 10 weeks for a single measurement. The distribution of gestation compared for the 7476 SFH measurements in the 2437 women presented here (figure 1*a*) was, as expected, larger than that observed by Linasmata in 1295 SFH measurements in 451 women from Bangkok [42] and in the 1498 SFH in 321 women from Prachuap, one of the central provinces in Thailand [53]. Growth curves of SFH against gestational age can vary by country as reported by Challis from a comparison of 11 studies of SFH measurements and ethnic group [27]. This would imply that model 3 with the parameter estimates for the population presented here may not be applicable to other countries, but the model itself could be re-parametrized for another country by using the data that are normally used to produce SFH growth curves and repeating the estimation process described here (that is training the model to a new dataset from a different population). In addition, it is likely to give more accurate predictions than other methods that use SFH. Thus, while this model is produced for refugee and migrant, predominantly Karen women of Asian origin, it has the potential to be adapted to other groups.

The multiple-measures model (model 3) predicts gestational age from SFH with consistently higher accuracy than other methods. The multiple-measures model was compared with linear and nonlinear models using the same dataset and was found to provide more accurate results using seven criteria (electronic supplementary material). The accuracy of model 3 applied to the dataset presented here was also compared with previously published methods applied to other datasets. The method by Andersson & Bergtrom [26] resulted in 45 per cent (270/604) cases delivered within two weeks of the predicted date, whereas model 3 gives 62 per cent delivery within two weeks of the predicted date. The method used by Faustin *et al*. [51] resulted in an average deviation from the real gestational age of two to three weeks, whereas for model 3 this deviation was less than one week. Reading gestational age from growth curves with fifth and 95th percentiles or 10th and 90th percentiles tends to result in accuracies of within five to eight weeks (i.e. ±2.5 to four weeks) [27,42,52], whereas the multiple-measures model predicts with an accuracy of within four weeks (95% prediction interval) when six or more SFH measurements are used. The reason why the multiple-measures model tends to predict gestational age with a higher accuracy than other SFH methods is because it incorporates not only the height measurements but also the slopes (gradients) between them. This allows the shape of the curve to be accounted for in terms of the relationship between the SFH and the growth velocity, a measure that has been shown in other recent studies using US to be highly informative [54].

The multiple-measures model should not be used to predict a binary variable such as prematurity (that is to predict whether the birth will be either premature or not premature). The reason for this is that the range of gestational ages of premature births is about five weeks and this is very close in size to the prediction interval, which at best is about four weeks. Thus, it is expected that many births on the border between term and preterm would be misclassified using the multiple-measures model. However, the model prediction is reliable as a continuous variable. This method is also robust to other risk factors including mother weight, mother height, mother BMI, mother age, the gravida of the current pregnancy, the parity of the current pregnancy, whether the mother smoked or not, slide positive for *P. falciparum* and slide positive for *P. vivax*.

In summary, given a realistic number (6–7) of repeated SFH measurements, at least two weeks apart, with corresponding dates derived from routine ANC, the multiple-measures model has the potential to predict gestational age to a higher level of accuracy than previously published methods. It can be applied to the presented population using the freely available Excel spreadsheet. Entry of a series of SFH measurements and the corresponding dates in this spreadsheet will generate a prediction of the date of birth with corresponding accuracy. The model could also be applied to other populations after training to the same data that were used to obtain SFH growth curves and development of a new spreadsheet for predictive purposes could be derived. The application of the model to different populations, particularly those with a different ethnicity will be the subject of future work.

The ideal of US dating for pregnant women worldwide will continue to be constrained by the available resources. The cost of SFH measurements and a computer to calculate EDD are orders of magnitude lower than the cost of an US machine. US performs better if the dating is in the optimum window, whereas SFH allows more flexibility. The study of infectious diseases in pregnancy in resource-limited settings needs appropriate technology. Multiple SFH measurements with an appropriate model for inferring gestational age is one such tool [55].
